# Efficacy and safety of tocilizumab in COVID-19 patients

**DOI:** 10.18632/aging.103988

**Published:** 2020-10-08

**Authors:** Kai-Lian Zheng, Ying Xu, Yu-Feng Guo, Le Diao, Xiang-Yu Kong, Xiao-Jian Wan, Feng Zhao, Fang-Zheng Ning, Li-Bing Wang, Fan Qiao, Jiang-Man Zhao, Jia-Huan Zhou, Yue-Qian Zhong, Shou-Xin Wu, Yi Chen, Gang Jin, Yu-Chao Dong

**Affiliations:** 1 Department of General Surgery, Changhai Hospital, Naval Medical University, Shanghai 200433, China; 2 Huoshenshan Hospital, Wuhan 430100, Hubei, China; 3 Department of Gastroenterology, Hankou Hospital, Wuhan 430000, Hubei, China; 4 Office of Medical Education, Naval Medical University, Shanghai 200433, China; 5 Shanghai Zhangjiang Institute of Medical Innovation, Shanghai Biotecan Pharmaceuticals Co., Ltd., Shanghai 201204, China; 6 Department of Gastroenterology, Changhai Hospital, Naval Medical University, Shanghai 200433, China; 7 Faculty of Anesthesiology, Changhai Hospital, Naval Medical University, Shanghai 200433, China; 8 Department of Cardiovascular Medicine, Changhai Hospital, Naval Medical University, Shanghai 200433, China; 9 Department of Hematology, Changhai Hospital, Naval Medical University, Shanghai 200433, China; 10 Department of Cardiac Surgery, Changhai Hospital, Naval Medical University, Shanghai 200433, China; 11 Department of infectious diseases, Changhai Hospital, Naval Medical University, Shanghai 200433, China; 12 Respiratory and Critical Care Medicine Department, Changhai Hospital, Naval Medical University, Shanghai 200433, China

**Keywords:** COVID-19, SARS-CoV-2, tocilizumab, interleukin-6, C-reactive protein

## Abstract

In this retrospective study we assessed the efficacy and safety of tocilizumab in patients with critical or severe coronavirus disease 2019 (COVID-19). We enrolled 181 patients admitted to Huoshenshan Hospital (Wuhan, China) with confirmed COVID-19 between January 2020 and February 2020. Ninety-two patients were treated with tocilizumab, and 89 patients were treated conventionally. We analyzed the clinical manifestations, changes in CT scan images, and laboratory tests before and after tocilizumab treatment, and compared these results with the conventionally treated group. A significant reduction in the level of C-reactive protein was observed 1 week after tocilizumab administration. In some cases this meant the end of the IL-6-related cytokine storm. In addition, tocilizumab relieved fever, cough, and shortness of breath with no reported adverse drug reactions. These findings suggest tocilizumab improves clinical outcomes and is effective for treatment of patients with critical or severe COVID-19. However, future clinical trials are needed to better understand the impact of tocilizumab interference with IL-6 and provide a therapeutic strategy for treatment of COVID-19.

## INTRODUCTION

Severe acute respiratory syndrome coronavirus (SARS-CoV), Middle East respiratory syndrome-related coronavirus (MERS-CoV), and severe acute respiratory syndrome coronavirus 2 (SARS-CoV-2) are zoonotic coronaviruses that cause upper respiratory tract infections. Although SARS-CoV-2 is less deadly, it is more transmissible, leading to greater outbreaks [1–4]. According to the World Health Organization (WHO), at least 212 countries and regions have reported confirmed cases of coronavirus disease 2019 (COVID-19) since the first case was recorded in the city of Wuhan. This has resulted in more than 337,736 deaths worldwide as of May 24, 2020 [5]. Most patients develop pneumonia with abnormal findings on chest computed tomography (CT), which is often followed by rapid deterioration to respiratory failure [6–8]. COVID-19 is clinically manifested by fever, cough, and dyspnea leading to respiratory failure [4]. The mortality rate of severe patients is high, and the increase in severe cases puts tremendous pressure on intensive care units (ICUs) and medical staff [6, 9]. Unfortunately, there is currently no effective treatment or antiviral medication for COVID-19 [4, 10].

Recent studies have shown that an increase in serum levels of proinflammatory cytokines, including interleukins (IL)-6, tumor necrosis factor α (TNF-α), and IL-12, is associated with the pulmonary inflammation and extensive lung damage previously seen in SARS-CoV and MERS-CoV infections, and currently in COVID-19 [6, 11–14]. Earlier studies showed that patients infected with MERS, SARS-CoV, and SARS-CoV-2 exhibited high plasma levels of IL-2, IL-6, IL-10, IFN-γ, granulocyte colony-stimulating factor (G-CSF), interferon-γ-inducible protein (IP-10), and TNF-α. This suggests that cytokine storms contribute to the severity and poor prognosis in these diseases [4, 6, 14–16] and may be an important factor affecting COVID-19 patient mortality.

IL-6 plays a central role in acute inflammation and cytokine storms [17], and increasing evidence suggests interference with IL-6 has a potential therapeutic effect in COVID-19 [4]. Tocilizumab is a humanized anti-IL-6 receptor (IL-6R) monoclonal antibody used to treat rheumatoid arthritis. By binding to both soluble IL-6R (sIL-6R) and membrane-bound IL-6R (mIL-6R), tocilizumab inhibits IL-6 signaling [18]. The results of long-term toxicity tests on animals showed that tocilizumab is well tolerated, and no significant abnormalities were observed in other clinicopathological studies or histopathological evaluations [19–21]. In 5 phase III double-blind controlled trials, in which treatment of rheumatoid arthritis was evaluated, there were no complications or disease deterioration or death related to tocilizumab [16]. Moreover, in a recent survey of 21 critical or severe COVID-19 patients, the results showed that tocilizumab is an effective treatment for severe COVID-19 patients [4]; however, the number of patients in the survey was small, and more data are still needed. In the presents study, we assessed the clinical features, laboratory characteristics, treatment, and clinical outcomes to determine the efficacy and safety of tocilizumab in COVID-19 patients.

## RESULTS

### Demographics and clinical characteristics

Table 1 shows the clinical characteristics of 181 COVID-19 patients, including 92 treated with tocilizumab and 89 conventionally treated patients. The mean ages of the tocilizumab and conventionally treated patients were 68.8 (range, 25-87) and 66.8 (range, 25-85) years, respectively. Among those receiving tocilizumab, 65 (70.7%) had at least one coexisting disorder (i.e., hypertension, diabetes, cardiovascular and/or cerebrovascular disease, cancer, or liver cirrhosis), whereas only 25 (28.1%) conventionally treated patients had a coexisting disorder (P<0.0001). Among tocilizumab treated patients, 84.8% had critical or severe disease before tocilizumab, whereas only 46.1% of those receiving conventional treatment were critical or severe (P< 0.0001). Conventionally treated patients had significantly fewer hospitalization days than the tocilizumab group (mean, 16.4 vs. 27.5, P<0.0001). A total of 83 (90.2%) patients receiving tocilizumab and 88 (98.9%) conventionally treated patients were discharged from the hospital. Nine patients in the tocilizumab-treated patients died, whereas only 1 conventionally treated patient died. There were no significant differences in age, sex distribution, and negative-conversing days between the two groups.

**Table 1:** Clinical characteristics of 181 patients with COVID-19 caused by SARS-CoV-2, according to disease severity.

**Clinical characteristics Mean (range)**	**Tocilizumab treatment (n=92)**	**Conventional treatment (n=89)**	**P**
Age, mean (range), years	68.8 (25-87)	66.8 (25-85)	0.258
Gender			0.213
Male	57/92(62.0%)	47/89 (52.8%)	
Female	35/92(38.0%)	42/89 (47.2%)	
Coexisting disorders	65/92(70.7%)	25/89 (28.1%)	0.001
State of illness			0.001
Critical or severe	78/92 (84.8%)	41/89 (46.1%)	
Moderate	14/92 (15.2%)	48/89 (53.9%)	
Hospitalization days (range) – days	27.5 (6-62)	16.4 (4-46)	0.001
Clinical outcome			0.018
Discharge from hospital	83/92 (90.2%)	88/89 (98.9%)	
Dead	9/92 (9.8%)	1/89 (1.1%)	
Negative-conversing, mean (range), days	22.4 (1-57)	18.8 (1-57)	0.184

Although fever, cough, shortness of breath, and fatigue were the initial symptoms of most patients, some patients developed chest distress, myalgia, and rhinorrhea. Atypical symptoms, such as diarrhea and headache, were less common and may have been associated with central nervous system [22] and digestive system invasion by SARS-CoV-2. After 1-3 days of tocilizumab treatment, a large portion of the COVID-19 patients showed clinical remission of their symptom (Table 2). We performed a logistic regression analysis to investigate whether COVID-19 severity and coexisting disorders of patients could affect the response to treatment. The results showed the critical patients were at a higher risk (OR=7.000, P=0.001) of showing no improvement in their cough than moderate and severe patients (Table 2). But it appears that the severity of the disease and coexisting diseases had no significant effect on other clinical symptoms.

**Table 2:** Clinical symptoms and influence on non-improvement of symptoms after tocilizumab treatment by COVID-19 severity and coexisting disorders.

**Clinical symptoms**	**Incidence rate at hospitalization**	**Improvement rate after tocilizumab treatment**	**Influence on no improvement of symptoms**			
			**Severity of COVID-19 (Critical vs. moderate & severe)**		**Coexisting disorders (Yes vs. No)**	
			**OR (95% CI)**	**P**	**OR (95% CI)**	**P**
Fever	67.4% (62/92)	61.3% (38/62)	3.200 (0.963-10.639)	0.058	0.648 (0.200-2.099)	0.469
Cough	77.2% (71/92)	71.8% (51/71)	7.000 (2.220-22.070)	0.001	0.798 (0.254-2.509)	0.700
Shortness of breath	68.5% (63/92)	60.3% (38/63)	0.843 (0.278-2.554)	0.762	1.846 (0.559-6.099)	0.315
Fatigue	42.4% (39/92)	59.0% (23/39)	2.800 (0.691-11.344)	0.149	0.463 (0.102-2.097)	0.318
Chest distress	37.0% (34/92)	61.8% (21/34)	1.250 (0.296-5.272)	0.761	1.200 (0.292-4.928)	0.800
Myalgia	17.4% (16/92)	50% (8/16)	/	/	/	/
Diarrhea	2.2% (2/92)	50% (1/2)	/	/	/	/
Headache	2.2% (2/92)	50% (1/2)	/	/	/	/
Rhinorrhea	1.1% (1/92)	100% (1/1)	/	/	/	/

### Laboratory indices

Table 3 summarizes the laboratory indices of 181 patients. After tocilizumab treatment, white blood cell counts, C-reactive protein (CRP) levels, maximum temperature, minimum oxygen saturation, highest respiratory rate, and maximum heart rate were all significantly improved (P< 0.0001, P< 0.0001, P< 0.0001, P< 0.0001, P=0.0011, P< 0.0001, respectively), and the mean values were close to normal. Before treatment, CRP levels were higher in tocilizumab-treated than conventionally treated patients (50.8±67.3 mg/L vs. 23.0±28.7 mg/L) (Table 3). The mean IL-6 level before tocilizumab was 102.1±535.1 pg/mL, indicating that IL-6 was upregulated in these COVID-19 patients. Notably, after 1 week of tocilizumab treatment, IL-6 levels in these patients’ had increased to 369.7±778.6 pg/mL (P< 0.0001). In sharp contrast, the mean IL-6 level before conventional treatment was only 29.2±71.6 pg/mL.

**Table 3:** Comparison of laboratory findings between tocilizumab and conventional treatment.

**Laboratory findings mean mean ± SD**		**Tocilizumab treatment**			**Conventional treatment**		
		**Before the tocilizumab**	**One week after tocilizumab treatment**	**P**	**Prior to admission**	**One week after hospitalization**	**P value**
White-cell count, ×10^9^/L		7.6±3.7	6.6±5.7	< 0.0001	7.2±3.9	7.0±3.8	0.571
	<4*10^9 /L	7/92	19/92		11/89	3/89	
	>10*10^9 /L	19/92	7/92		8/89	6/89	
Absolute lymphocyte, ×10^9^/L		1.4±3.3	1.3±0.6	0.1754	1.4±0.6	1.5±0.7	0.008
	<1.1*10^9 /L	43/92	29/92		36/89	15/89	
C-reactive protein, mg/L		50.8±67.3	7.0±22.2	< 0.0001	23.0±28.7	21.0±34.7	0.428
	>7 mg/L	70/92	14/92		51/89	31/89	
Procalcitonin, ng/ml		0.5±2.5	0.2±0.5	0.0216	0.4±1.8	0.1±0.2	0.326
	>0.05 ng/mL	52/92	21/92		36/89	17/89	
Interleukin-6, pg/mL		102.1±535.1	369.7±778.6	< 0.0001	29.2±71.6	29.4±81	0.219
	>7 pg/mL	86/92	91/92		76/89	69/89	
Maximum temperature, °C		37.4±0.7	36.9±0.4	< 0.0001	36.7±0.6	37.1±0.4	< 0.0001
Minimum oxygen saturation, %		90.3±8.0	93.1±9.9	< 0.0001	96.0±2.9	94.1±4.8	< 0.0001
Neutrophils, ×10^9^/L		5.7±3.4	4.4±5.4	< 0.0001	4.8±3.3	4.7±3.6	0.265
	>6.3 *10^9 /L	34/92	11/92		16/89	11/89	
Total bilirubin, umol/L		11.9±6.4	10.8±10.1	0.0026	11.6±12.9	12.6±19.9	0.847
	>20.5 umol/L	9/92	7/92		5/89	4/89	
Direct bilirubin, umol/L		5.0±3.1	4.1±4.7	0.0006	5±8.2	5.6±11.9	0.914
Highest respiratory rate		29.2±14.4	26.2±19.7	0.0011	20.3±2.5	22.3±2.1	< 0.0001
Maximum heart rate		109±21.1	95.5±14.9	< 0.0001	92.1±14.8	102.8±15.6	< 0.0001
Platelet count, *10^9 /L		220±86.9	219.2±83	0.5118	228.3±96	246.5±91	0.100
	<125*10^9 /L	14/92	5/92		10/89	6/89	
Mean platelet volume		10.3±1.2	9.9±1.1	0.0730	10.1±1.2	9.9±1.1	0.078
Total protein, g/L		61.0±6.9	62.4±7.1	0.2706	65.3±7.1	64.2±9.6	0.881
	<60 g/L	46/92	28/92		20/89	16/89	
Albumin, g/L		33.0±4.7	35.4±4	0.0009	36±4.1	35.8±4.7	0.537
	<34 g/L	54/92	28/92		23/89	28/92	
Alanine aminotransferase		35.3±29.6	47.6±87.9	0.5240	27.1±26.6	30±29.9	0.499
Aspartate aminotransferase		26.6±14.3	35.3±57.9	0.3393	29.9±44.3	27.5±25.4	0.361
Ratio of albumin to globulin		1.2±0.2	1.3±0.3	0.0177	1.2±0.2	1.2±0.2	0.739
Lactate dehydrogenase		261.9±111.3	241.1±82.3	0.3729	205.4±83.5	197.4±47.7	0.079
Carbon dioxide combining power		23.8±3.3	24.7±2.8	0.077	24.3±2.5	27.5±20	0.214
Creatinine, umol/L		78.5±41.9	75.9±36.7	0.888	70±28.4	68.6±25.8	0.001
Blood urea nitrogen, mmol/L		6.2±3.7	10.4±34.1	0.8074	5.5±2.9	6±2.7	0.992
Sodium, mmol/L		138.1±14	140±3.3	0.2887	141.5±4.7	140.9±3.8	0.291
Potassium, mmol/L		4.2±0.5	4.4±0.4	0.2087	4.1±0.5	4.2±0.5	0.476
Calcium, mmol/L		2.0±0.1	2.1±0.1	< 0.0001	2.1±0.1	2.2±0.1	0.054
Hypersensitive troponin, ng/L		0.1±0.3	0.03±0.08	0.3792	0±0.2	0±0.1	0.203
Fibrinogen, g/L		3.7±1.3	2.5±0.7	< 0.0001	3.4±0.7	3.6±2.1	0.437
D-dimer, mg/L		2.5±3.3	2.9±3.7	0.1929	1.3±1.6	1.4±1.2	0.069

### Imaging features

All patients had abnormal chest CT on presentation before tocilizumab treatment. The primary abnormalities on the initial chest CT were plaque-like, ground-glass opacities and focal consolidation. However, after 1 week of tocilizumab treatment, most CT scans showed significant remission.

### Clinical presentations

We evaluated the effects of tocilizumab treatment 1 week after treatment. We found that 72 patients showed improvement, 10 patients showed no improvement, and 9 critical patients had died. We also evaluated the effects of conventional treatment within 2 weeks of hospitalization. We found that 79 patients had improved, 9 patients showed no improvement, and 1 critical patient had died. In that patient, IL-6 level suddenly rose from 529.5 pg/mL to 5000 pg/mL before his death. This result suggests that in critical and severe patients, IL-6 levels may determine the prognosis and overall mortality of COVID-19 patients. We therefore focused our investigation on factors affecting early-stage clinical outcomes of severe patients.

### Treatment

Immediately before and within 2 weeks after hospitalization, IL-6 levels in the 89 conventionally treated patients exhibited little change (mean, 29.2±71.6 pg/mL vs. 29.4±81 pg/mL, P=0.219). In sharp contrast, after 3 days of treatment, IL-6 levels in the 92 tocilizumab-treated patients had increased significantly (102.1±535.1 pg/mL vs. 337.7±723.8 pg/mL, P< 0.0001), while white blood cell counts had significantly decreased (7.6±3.7 ×10^9^/L vs. 6.6±5.7 ×10^9^/L, P< 0.0001). In addition, CRP levels were significantly lower 1 week after tocilizumab treatment (50.8±67.3 mg/L vs. 7.0±22.2 mg/L, P< 0.0001) (Table 3).

**Figure 1:**
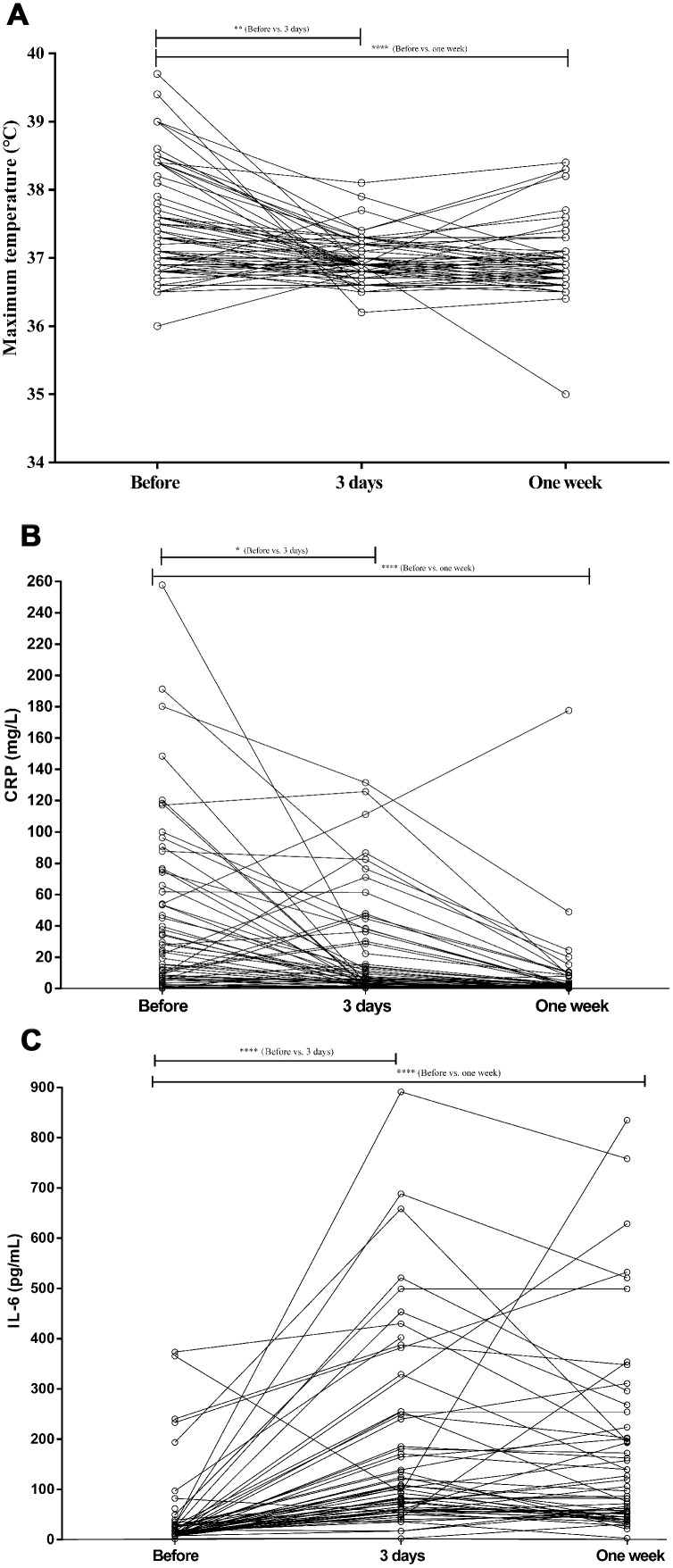
**The values of maximum temperature, CRP, and IL-6 levels before and after tocilizumab treatment in COVID-19 patients.** (**A**) The fever returned toward normal in most patients treated conventionally or with tocilizumab. (**B**) CRP levels decreased significantly after tocilizumab treatment and returned to normal in most conventionally treated patients. (**C**) IL-6 increased significantly after tocilizumab treatment in most patients.

Three days after tocilizumab treatment, the body temperature of most patients had dropped, gradually moving closer to the normal range (Figure 1A). Simultaneously, the clinical symptoms were greatly attenuated over the following days. Figure 1B shows that 1 week after tocilizumab treatment, the CRP levels had decreased significantly in all but one patient. In that patient, who was critical, CRP levels greatly increased, and the patient died after 27 days in the hospital. Intriguingly, IL-6 levels increased significantly in tocilizumab-treated patients, though their condition was significantly improved (Figure 1C). No adverse drug reactions were reported during the tocilizumab treatment.

### Risk factors for poor clinical outcomes in critical COVID-19 patients

Univariate logistic regression analysis showed that high maximum temperature (OR=5.205 [95% CI, 1.160-23.358], P=0.031), maximum heart rate (OR=1.088 [95% CI, 1.010-1.171], P=0.026), and lactate dehydrogenase levels (OR=1.023 [95% CI, 1.004-1.043], P=0.018) were risk factors for poor clinical outcomes among critical patients before tocilizumab treatment. In addition, maximum heart rate (OR=1.169 [95% CI, 1.007-1.357], P=0.041), white blood cell count (OR=1.503 [95% CI, 1.029-2.196], P=0.035), mean platelet volume (OR=2.902 [95% CI, 1.040-8.100], P=0.042), blood urea nitrogen (OR=1.426 [95% CI, 1.048-1.941], P=0.024), sodium (OR=1.488 [95% CI, 1.027-2.158], P=0.036), and CRP (OR=1.082 [95% CI, 1.003-1.168], P=0.041) were all risk factors for poor clinical outcomes among critical patients 1 to 3 days after tocilizumab treatment. High minimum oxygen saturation (OR=0.704 [95% CI, 0.498-0.995], P=0.047) and high platelet count (OR=0.979 [95% CI, 0.958-0.999], P=0.042) were identified as protective factors against poor clinical outcomes among severe patients. Conversely, reduced platelet count was a risk factor for poor clinical outcomes (Table 4).

**Table 4:** Univariate logistic regression analysis to investigate risk factors for poor diagnosis of critical COVID-19 patients.

**Variables**	**OR (95% CI)**	**P**
**Before treatment**		
Maximum temperature, °C	5.205 (1.160-23.358)	0.031
Maximum heart rate	1.088 (1.010-1.171)	0.026
Lactate dehydrogenase	1.023 (1.004-1.043)	0.018
**1-3 days after tocilizumab treatment**		
Minimum oxygen saturation, %	0.704 (0.498-0.995)	0.047
Maximum heart rate	1.169 (1.007-1.357)	0.041
White-cell count, ×10^9/L	1.503 (1.029-2.196)	0.035
Platelet count, *10^9 /L	0.979 (0.958-0.999)	0.042
Mean platelet volume, fL	2.902 (1.040-8.100)	0.042
Blood urea nitrogen, mmol/L	1.426 (1.048-1.941)	0.024
Sodium, mmol/L	1.488 (1.027-2.158)	0.036
C-reactive protein, mg/L	1.082 (1.003-1.168)	0.041

## DISCUSSION

SARS-CoV-2, the virus that causes COVID-19, as well as SARS-CoV and MERS-CoV belong to genus β and infect the lower respiratory tract, causing severe respiratory syndrome in humans [23, 24]. On May 25, 2020, the Chinese Center for Disease Control and Prevention reported 84,536 confirmed COVID-19 cases and 4,645 related. Thus COVID-19 had caused more deaths than severe acute respiratory syndrome (SARS) or MERS [25].

In this retrospective study, we observed a cohort of 181 COVID-19 patients with laboratory-confirmed SARS-CoV-2 infections. This included 92 patients treated with tocilizumab and 89 treated conventionally. In a survey of 21 critical or severe COVID-19 patients, the clinical data showed that, in most patients, respiratory symptoms, hypoxemia, and CT opacity changes improved immediately after tocilizumab treatment, which suggests tocilizumab may be a efficacious treatment for COVID-19 [4]. In the present study, analysis of the tocilizumab-treated patients yielded similar results. However, after comparison with the outcomes of the conventionally treated patients, we believe that the patient's own immunity, disease severity, and comprehensive treatment are directly related to the outcomes with these patients. They cannot be attributed solely to tocilizumab treatment. Nonetheless, it is undeniable that tocilizumab, an IL-6 blocker, played an important role in alleviating cytokine storms, which can significantly improve the clinical symptoms and laboratory findings of critical and severe patients.

Consistent with earlier reports [4], no adverse drug reactions or subsequent pulmonary infection were reported with tocilizumab treatment. The observation of dynamic IL-6 levels also helps to confirm the COVID-19 response to treatment [26]. Consistent with earlier observations [26], we found that IL-6 levels spiked after tocilizumab treatment. This is likely because IL-6 is mainly eliminated via IL-6R-mediated clearance [27], and the binding of tocilizumab to IL-6R inhibits receptor-mediated IL- 6 clearance [26]. Primarily synthesized by liver hepatocytes and secreted in plasma, CRP is regulated mainly by IL-6 [28]. In our study, CRP levels were elevated in 76.1% of patients (70/92) before receiving tocilizumab, but this was reduced to 15.2% (14/92) 1 week after tocilizumab treatment, indicating the patient's inflammation was alleviated. Correspondingly, the patients’ clinical symptoms also significantly improved after tocilizumab. It therefore appears that tocilizumab can be used to effectively treat patients with COVID-19, and that its beneficial effects are related to its ability to suppress IL-6-related febrile and inflammatory storm responses [4]. In addition, we observed the IL-6 levels in some patients were trending downward 1 week after tocilizumab treatment, which may also reflect the inhibitory effect of tocilizumab on inflammatory activity and the resultant improvement in the patients’ clinical status.

As a virus spreads within a host, the host’s immune response is activated, and an environment of cytokines is established, which can lead to clearance of the virus and cure for the patient [29]. However, when cytokines increase past a threshold level, they can trigger a cytokine storm, which can severely damage multiple organs and lead to multiple organ dysfunction syndrome or death [9, 29–31]. In the treatment of severe and critical COVID-19 patients, the balance between immune activation and inflammation inhibition is crucial. The latest research shows that any preventive intervention that reduces inflammation may have a negative effect on viral clearance [29]. In the present study, there was no significant difference in negative-conversing days between tocilizumab-treated and conventionally treated patients (22.4 vs. 18.8, P=0.184). The dose and frequency of tocilizumab administration were in accordance with Chinese management guidelines for COVID-19 (version 7.0) [32]. Most patients with COVID-19 were administered the drug only once, and the total amount did not exceed 400 mg. Based on our findings, we believe the appropriate amount of tocilizumab to reduce inflammation is beneficial, and does not affect the number of negative-conversing days in COVID-19 patients. This suggests the appropriate level of tocilizumab treatment may not negatively affect viral clearance. However, more clinical research on IL-6 and other cytokines is needed to determine the proper balance between immune activation and inflammation inhibition.

Our univariate and multivariate regression analyses showed maximum temperature to be an independent risk factor for poor clinical outcomes of critical patients before tocilizumab treatment. Then 1-3 days of after tocilizumab treatment, mean platelet volume and CRP were identified as independent risk factors for poor clinical outcomes among critical patients. Mean platelet volume is an indicator of inflammation in patients with various diseases [33–35]. Cytokine storms mediated by overproduction of proinflammatory cytokines is an important factor leading to high mortality in COVID-19 patients and may be a key reason for their poor prognosis. CRP is a nonspecific acute-phase protein induced by IL-6 and a sensitive biomarker of inflammation and infection [36]. During acute inflammatory responses, CRP expression rapidly increases [37, 38]. Recent research showed that the serum CRP levels can be used to effectively assess disease severity in COVID-19 patients [39]. Therefore, an increase in CRP may also be a sign of poor patient prognosis.

This study has several limitations, including the small number of patients, the need to enhance the evidence strength, and missing viral load data. Additionally, other cytokines, including IL-2, IL-10, and TNF-α, should also be considered.

Under its medication guidelines, tocilizumab can effectively improve clinical symptoms and slow the deterioration of COVID-19 patients. However, directly related to any patient’s prognosis is also the immunity of the patient, the severity of the disease, and the comprehensive treatment. Although these preliminary results look promising, more evidence and high-quality clinical trials are required to understand the efficacy of tocilizumab in COVID-19. And of course the development of vaccines will be the most effective way to prevent COVID-19.

## MATERIALS AND METHODS

### Study population

This single-center, retrospective observational study recruited 181 patients admitted to Huoshenshan Hospital (Wuhan, China) with confirmed COVID-19 between January 2020 and February 2020. Ninety-two patients were treated with tocilizumab, and 89 patients were treated with conventional treatment. All patients were diagnosed with COVID-19 based on World Health Organization interim guidance, and were clinically classified based on the Chinese management guidelines for COVID-19 (version 7.0) [32]. Moderate patients were defined as those with fever, respiratory symptoms and imaging evidence of pneumonia. Patients who met any of the following criteria were defined as severe: 1) tachypnoea (≤30 breaths/ min), 2) oxygen saturation ≤93% at rest, 3) PaO_2_/FIO_2_ <300 mmHg (1 mm Hg=0.133 kPa), and 4) progression of the lesion by >50% within 24-38 hours based on pulmonary imaging. Critical patients were defined as those with one or more of the following criteria: 1) respiratory failure, 2) septic shock, or 3) multiple organ dysfunction and/or failure.

According to the Chinese management guideline for COVID-19 (version 7.0) [32], the standard ages for patients who can be treated with tocilizumab are between 18 and 85 years. In addition, these patients must exhibit elevated IL-6 levels and multiple lung lesions showing significant progression of >50% within 24-38 hours on pulmonary imaging. The first dose of tocilizumab was 4-8 mg/kg. The recommended dose is 400 mg. A total of 100 ml of saline was added, and the infusion time was more than 1 hour. For patients presenting with fever, an additional dose (same dose as before) was administered if there was still fever after 24 hours. The interval between the two administrations was always ≥12 hours, and the cumulative number of doses was never more than 2. The maximum single dose never exceeded 800 mg.

Tocilizumab was contraindicated for pregnant or lactating women; patients with ALT/AST >5 times ULN, neutrophils <0.5×10^9^/L, platelets less than 50×10^9^/L; patients diagnosed of rheumatic immune-related disease; patients on long-term oral anti-rejection drugs or immunomodulatory drugs; patients hypersensitive to tocilizumab or any excipients; patients with active tuberculosis, hepatitis, and definite bacterial and fungal infections; organ transplant patients; and patients with mental disorders. IL-6 levels were measured using FACS or electrochemical luminescence methods (Roche Diagnostics GmbH). The normal range of IL-6 was <7 pg/mL.

The study was approved by Medical Research Ethics Committee of the Huoshenshan Hospital (Approval number: hssll033). All patients provided informed consent before treatment and agreed to publication of this case series. We are committed to protecting patient privacy and complying with the Helsinki Declaration.

### Procedure

Demographic, clinical, laboratory, and treatment data were collected from electronic medical records. All data were checked by two physicians. The personal and clinical data collected included sex, age, medical history, and initial symptoms, including fever, cough, shortness of breath, fatigue, chest distress, myalgia, diarrhea, headache, and rhinorrhea. Real-time reverse transcriptase-polymerase chain reaction assays were used to detect SARS-CoV-2 infection. In addition, patients underwent an initial examination and then complete blood counts (white blood cells, neutrophils, lymphocytes, and platelets), serum biochemical tests (renal and liver function, carbon dioxide combining power, creatinine, and electrolytes), coagulation profile, procalcitonin, CRP, and myocardial enzymes 1-3 days and 1 week after tocilizumab. The patients’ follow-up endpoint was discharge or death. CT scans were used to image the lungs of all patients. The effect of treatment on COVID-19 patients after hospitalization for 1 week was evaluated based on Chinese management guidelines for COVID-19 (version 7.0) [32], as described previously.

### Statistical analysis

Statistical analysis were performed using SPSS 19.0 (IBM, NY, USA) and R 3.5.1 software. Continuous variables are presented as the mean (range) or mean ± SD. If continuous variables conformed to a normal distribution, unpaired *t*-tests were used to analyze differences. Otherwise, the Mann-Whitney U test was used. When comparing datasets containing multiple groups, one-way analysis of variance was used for normally distributed datasets, and the Kruskal-Wallis test was used for datasets not normally distributed. Categorical variables were summarized as the counts and percentages, and analyzed using the χ^2^ test or Fisher’s exact test, as appropriate. Univariate logistic regression analysis were performed to identify risk factors for disease progression. A two-sided values of P<0.05 were considered statistically significant.
